# Severe Aplastic Anemia Developed after Thymectomy: A Case Report and Literature Review

**DOI:** 10.1155/2020/7819321

**Published:** 2020-01-13

**Authors:** Bruna Cristine Duarte Rodrigues, Pedro José Galvão Freire, Beatriz Pinto e Siqueira Campos, Juliana Oliveira Vieira, Pedro Alves da Cruz Gouveia

**Affiliations:** ^1^Internal Medicine Service, Federal University of Pernambuco, Recife, PE, Brazil; ^2^Hematology Service, Federal University of Pernambuco, Recife, PE, Brazil

## Abstract

Thymus neoplasms are frequently related to paraneoplastic autoimmune manifestations. Its most common associations are myasthenia gravis and pure red cell aplasia. Aplastic anemia has been increasingly documented as an initial presentation of thymoma. Nevertheless, its development after successful surgical resection of thymoma is a rare condition. We report a case of a 53-year-old man with severe aplastic anemia preceded by amegakaryocytic thrombocytopenia three years after thymectomy with no signs of disease recurrence. He underwent immunosuppressive therapy with cyclosporine 5 mg/kg/day and prednisone 2 mg/kg/day for six weeks. Considering the availability of a compatible donor, allogeneic stem cell transplantation was carried out. However, the patient died 11 days after transplant. A literature review was conducted, and another ten cases of aplastic anemia, diagnosed three months to four years after thymectomy, were identified. These cases suggest persistence of peripheral self-reactive T lymphocytes even years after tumor definitive treatment.

## 1. Introduction

Thymus neoplasms are known for their association with different paraneoplastic autoimmune manifestations. Its most common associations are myasthenia gravis and pure red cell aplasia [1, 2]. Other less frequently found hematologic complications are aplastic anemia, autoimmune hemolytic anemia, pernicious anemia, immune thrombocytopenic purpura, agranulocytosis, and hypogammaglobulinemia [3]. Although being an uncommon association, over the last years several case reports of aplastic anemia following thymecotymy have been published. However, development of such autoimmune manifestation after successful surgical resection of the tumor is rarely described in the literature [2–10]. We report a case of a 53-year-old man with severe aplastic anemia three years after thymectomy with no signs of disease recurrence.

## 2. Case Presentation

**Figure 1:**
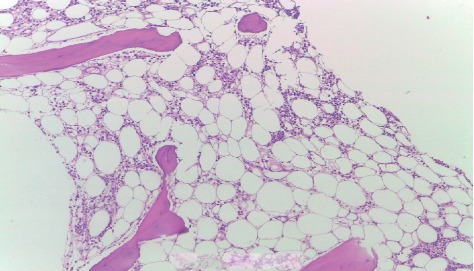
Fat-filled hypocellular bone marrow biopsy without fibrosis or anomalous cell infiltrate.

A 53-year-old man was admitted for investigation of dyspnea on moderate exertion, which started in the previous month. On admission, he presented with pancytopenia with a hemoglobin level of 3.4 g/dL, white blood count of 1960/mm³ without dysplastic changes (1270 lymphocytes and 520 neutrophils), and platelet count of 3120/mm³. Bone marrow biopsy showed 10% cellularity with hypoplasia of all hematopoietic series, predominantly myeloid series. There were no signs of anomalous cells, dysplastic changes, infiltration by malignant cells, or fibrosis. Histopathological findings were consistent with aplastic anemia (Figure 1).

The patient had previous history of asymptomatic mediastinal mass three years ago, diagnosed in preoperative exams for excision of gouty tophus on the elbow. The mediastinal tumor was resected, and pathological and immunohistochemical studies were compatible with encapsulated type B1 thymoma with free surgical margins. In the same year, throughout postoperative follow-up, isolated thrombocytopenia was identified. Bone marrow showed hypocellular megakaryocytic clustering without dysplasia and other series without alterations. These findings were consistent with amegakaryocytic thrombocytopenia, which progressed about three years to current admission pancytopenia.

Search for secondary causes of aplastic anemia was performed, and there was no exposure to drugs, radiation, or chemicals. Normal levels of folic acid and vitamin B12 were found, besides negative serology for hepatitis B, C, human immunodeficiency virus (HIV), and Epstein-Barr virus (EBV). Flow cytometry did not identify paroxysmal nocturnal hemoglobinuria (PNH) clones. Serum protein electrophoresis showed no monoclonal protein or hypogammaglobulinemia. Abdominal ultrasound showed no signs of visceromegaly.

In order to investigate active thymoma, chest computed tomography (CT) was performed, but no alterations were found. To completely rule out recurrence of thymoma, an Fluorine(18)-fluorodeoxyglucose PET/CT scan was performed and showed a physiological distribution of the radiotracer. Thus, aplastic anemia therapy was initiated with cyclosporine 5 mg/kg/day and prednisone 2 mg/kg/day. Antithymocyte globulin was not available to complement the therapy. Treatment was maintained for six weeks, given the availability of a compatible HLA donor. The patient underwent allogeneic stem cell transplantation; however, on the 11^th^ day after transplantation, he developed fusariosis and died of septic shock.

## 3. Discussion

Thymoma represents 20% of mediastinal neoplasms and highest prevalence in individuals between 40 and 60 years old, with similar incidence rates between both genders [11]. Its detection typically occurs when asymptomatic mediastinal masses, compressive symptoms, or paraneoplastic autoimmune manifestations are identified. In about 44% of cases, it is associated with myasthenia gravis, which leads to earlier diagnosis. On the other hand, when it presents with hematological paraneoplastic involvement, its identification tends to occur in later stages [12].

Pure red blood cell aplasia is the most common haematological abnormality, detected in about 15% of thymomas. Aplastic anemia is also an autoimmune disorder that is sometimes associated with thymoma [12]. In such cases, thymoma is usually diagnosed in etiological investigation of a previously diagnosed aplastic anemia [2, 13, 14]. However, the occurrence of this hematologic disease after thymectomy is uncommon as described in our case. Aplastic anemia may be preceded by isolated cytopenias such as amegakaryocytic thrombocytopenia reported here [1, 4, 6, 9].

A literature review was conducted in the PubMed database with English language case reports of patients who developed aplastic anemia after thymectomy. After reviewing publications from 1993 to 2018, we were able to identify a total of nine publications [2–10] with a total of ten cases that we summarize in Table 1. Six patients were male and four females. Pancytopenia was diagnosed between three months [5, 9] and four years [4] after thymectomy with an average of 19.6 months. In all cases, there were no signs of disease recurrence on CT scan; however, only the present case ruled out this possibility by also performing a PET/CT scan. Two patients were diagnosed with thymoma in the presence of signals and symptoms of myasthenia gravis [2, 6], while the other cases reported no systemic abnormalities other than hematologic ones. Regarding hematological progress, in one case aplastic anemia was preceded by immune thrombocytopenic purpura [4] and in three others by the association of pure red cell aplasia and amegakaryocytic thrombocytopenia [6, 9, 10]. The present case was only preceded by isolated amegakaryocytic thrombocytopenia. In all cases, except one whose treatment was not reported [6], the immunosuppressive regimen contained cyclosporin A and six demonstrated good response to immunosuppression [2–5, 7, 8]. Studies have demonstrated that increases in cell count may require different time intervals, besides exhibiting different follow-up times to evaluate disease recurrence. Most patients had an effective response to treatment; however, in two, the response was not described [6, 10]. In addition to our study, we were only able to identify one case where a patient underwent allogeneic stem cell transplantation and, in contrast to our case, long-term cure was documented [9].

Despite the association of thymoma with these autoimmune diseases, thymus resection may not completely reverse previously existing clinical manifestations. Specific treatment of these manifestations is often necessary even after thymectomy [7, 12]. This evolution corroborates the pathophysiology that was proposed by other studies suggesting the persistence of peripheral self-reactive T lymphocytes (originated from thymoma). These peripheral T lymphocytes could be responsible for promoting autoimmune phenomena, even at later times after curative thymectomy [2, 7, 8, 10]. We identified case reports of aplastic anemia developed three to 48 months after thymus resection [4, 5, 9].

Treatment of aplastic anemia after thymectomy usually requires prolonged immunosuppressive therapy and has a higher relapse rate after weaning off immunosuppressive therapy than other causes of aplastic anemia [2]. There are few reported cases of allogeneic stem cell transplantation in the treatment of autoimmune haematological diseases after thymectomy [9, 15]. This type of transplant should usually be the first line in the management of idiopathic aplastic anemia cases with good performance status and HLA-compatible donor, in patients aged under 50 years [16]. In the case reported here, antithymocyte globulin was not available and the patient had transplant conditions. However, the outcome was negative with the patient's death on the 11^th^ postoperative day.

As for amegakaryocytic thrombocytopenia, management guidelines are even scarcer. Case reports suggest good response to immunosuppression also recommended for aplastic anemia treatment. This cytopenia may be, as observed in our case, an early presentation of aplastic anemia [6, 10].

Here we report a case of a late autoimmune manifestation suggesting persistence of peripheral self-reactive T lymphocytes even years after a curative thymectomy. This condition can lead to the development of hematological diseases such as severe aplastic anemia described here. We would emphasize that this hematological condition may be preceded by other blood abnormalities such as amegakaryocytic thrombocytopenia. This case suggests patients with thymoma should have periodic evaluations of their blood cell count, aiming at early identification of the most varied hematological alterations.

## Figures and Tables

**Table 1:** Review of published cases of aplastic anemia after thymectomy.

Authors	Ref.	Sex/age	Treatment for thymoma	Interval between thymoma and AA	Progress of blood disorder	Associated diseases	Treatment for AA	Effect
Kobayashi et al.	[4]	F/63	TMT + RT	4 years	ITP ⟶ AA	ND	Splenectomy + CyA	E
Dinçol et al.	[5]	M/38	TMT	3 months	AA	ND	CyA + ATG + mPSL + G-CSF	E
Ritchie et al.	[2]	M/50	TMT + CT + RT	1 year	AA	Myasthenia gravis	mPSL + CyA + ATG	E
Park et al.	[3]	F/61	TMT	16 months	AA	ND	CyA	E
Maslovsky et al.	[6]	M/42	TMT + CT	12 months	PRCA + AT ⟶ AA	Myasthenia gravis	ND	ND
Bajel et al.	[7]	M/67	TMT	6 months	AA	ND	CyA + ATG	E
de Castro et al.	[8]	M/69	TMT	3 years	AA	ND	CyA	E
		F/59	TMT + RT	2 years	AA	ND	CyA + PDN	NE
Simkins et al.	[9]	F/61	CT + TMT	3 months	PRCA + AT ⟶ AA	ND	ATG + mPSL + CyA ⟶ SCT	NE/E
Dahal et al.	[10]	M/63	TMT + CT + RT	More than 3 years	PRCA + AT ⟶ AA	ND	CyA	ND
Present case	—	M/53	TMT	3 years	AT ⟶ AA	ND	CyA + PDN ⟶ SCT	NE

Ref.: reference; AA: aplastic anemia; age: age at diagnosis of AA; TMT: thymectomy; CT: chemotherapy; RT: radiotherapy; CyA: cyclosporin A; ITP: immune thrombocytopenia purpura; ATG: antithymocyte globulin; PDN: prednisone; mPSL: methylprednisolone; ND: not described; NE: not effective; E: effective; PRCA: pure red cell aplasia; AT: amegakaryocytic thrombocytopenia; AA: aplastic anemia; G-CSF: granulocyte-colony stimulating factor; SCT: stem cell transplantation.
